# Rationale for enteroviral vaccination and antiviral therapies in human type 1 diabetes

**DOI:** 10.1007/s00125-019-4811-7

**Published:** 2019-01-23

**Authors:** Jessica L. Dunne, Sarah J. Richardson, Mark A. Atkinson, Maria E. Craig, Knut Dahl-Jørgensen, Malin Flodström-Tullberg, Heikki Hyöty, Richard A. Insel, Åke Lernmark, Richard E. Lloyd, Noel G. Morgan, Alberto Pugliese

**Affiliations:** 1 JDRF, 26 Broadway, 14th Floor, New York, NY 10004 USA; 2 Institute of Biomedical and Clinical Science, University of Exeter Medical School, RILD Building, Barrack Road, Exeter, EX2 5DW UK; 3 Departments of Pathology and Pediatrics, University of Florida, College of Medicine, Gainesville, FL USA; 4 School of Women’s and Children’s Health, Faculty of Medicine, University of New South Wales, Sydney, NSW Australia; 5 Department of Pediatric and Adolescent Medicine, Oslo University Hospital, Oslo, Norway; 6 Institute of Clinical Medicine, Faculty of Medicine, University of Oslo, Oslo, Norway; 7 Center for Infectious Medicine, Department of Medicine Huddinge, Karolinska Institutet, Karolinska University Hospital, Stockholm, Sweden; 8 Faculty of Medicine and Life Sciences, University of Tampere, Tampere, Finland; 9 Fimlab Laboratories, Pirkanmaa Hospital District, Tampere, Finland; 10 Insel Consulting, LLC, Rochester, NY USA; 11 Department of Clinical Sciences, Lund University/CRC, Skåne University Hospital, Malmö, Sweden; 12 Department of Molecular Virology and Microbiology, Baylor College of Medicine, Houston, TX USA; 13 Diabetes Research Institute, Miller School of Medicine, University of Miami, Miami, FL USA; 14 Department of Medicine, Division of Endocrinology, Diabetes and Metabolism, Miller School of Medicine, University of Miami, Miami, FL USA; 15 Department of Microbiology and Immunology, Miller School of Medicine, University of Miami, Miami, FL USA

**Keywords:** Antiviral therapy, Autoimmunity, Beta cells, Enterovirus, Pancreas, Prevention, Type 1 diabetes, Vaccine, Virus

## Abstract

In type 1 diabetes, pancreatic beta cells are destroyed by chronic autoimmune responses. The disease develops in genetically susceptible individuals, but a role for environmental factors has been postulated. Viral infections have long been considered as candidates for environmental triggers but, given the lack of evidence for an acute, widespread, cytopathic effect in the pancreas in type 1 diabetes or for a closely related temporal association of diabetes onset with such infections, a role for viruses in type 1 diabetes remains unproven. Moreover, viruses have rarely been isolated from the pancreas of individuals with type 1 diabetes, mainly (but not solely) due to the inaccessibility of the organ. Here, we review past and recent literature to evaluate the proposals that chronic, recurrent and, possibly, persistent enteroviral infections occur in pancreatic beta cells in type 1 diabetes. We also explore whether these infections may be sustained by different virus strains over time and whether multiple viral hits can occur during the natural history of type 1 diabetes. We emphasise that only a minority of beta cells appear to be infected at any given time and that enteroviruses may become replication defective, which could explain why they have been isolated from the pancreas only rarely. We argue that enteroviral infection of beta cells largely depends on the host innate and adaptive immune responses, including innate responses mounted by beta cells. Thus, we propose that viruses could play a role in type 1 diabetes on multiple levels, including in the triggering and chronic stimulation of autoimmunity and in the generation of inflammation and the promotion of beta cell dysfunction and stress, each of which might then contribute to autoimmunity, as part of a vicious circle. We conclude that studies into the effects of vaccinations and/or antiviral drugs (some of which are currently on-going) is the only means by which the role of viruses in type 1 diabetes can be finally proven or disproven.

## Introduction

A role for infectious agents in type 1 diabetes was proposed in the 1920s, following reports of diabetes after parotitis [1], suggesting viruses with affinity for the pancreas existed. Since then, numerous studies have reported associations between type 1 diabetes and a variety of viruses, including enteroviruses, herpesviruses, parechoviruses, rotaviruses and retroviruses [2]. Of these, the most extensive studies and robust associations have been observed with enteroviruses, both clinically and experimentally. Nonetheless, a role for viruses in type 1 diabetes is still considered unproven. Here, we review the evidence and consider how viruses might contribute to type 1 diabetes in multiple, perhaps unconventional, ways. From this evidence, we propose that the development and clinical evaluation of an enterovirus vaccine for preventing type 1 diabetes is scientifically justified.

## Epidemiological and genetic studies implicate enteroviruses in type 1 diabetes

In 1969, seasonal variation in type 1 diabetes diagnoses was correlated with the prevalence of the enterovirus coxsackievirus B (CVB)4 [3]. In 1973, individuals with newly diagnosed type 1 diabetes were found to have neutralising antibodies to CVB4 more frequently than control individuals [4]. Since then, many other epidemiological and clinical investigations have been conducted in birth cohorts of children at genetic risk of type 1 diabetes to address the role of enteroviruses in the initiation and acceleration of islet autoimmunity [5–8]. The results have not always been concordant, in part due to methodological limitations and inadequacies of sampling timing and frequency; however, the weight of the evidence, derived from multiple countries, clearly supports an association between enteroviruses and type 1 diabetes [9, 10]. This is supported by studies reporting that: (1) maternal viral infection in pregnancy, including enteroviral, is linked to type 1 diabetes risk in the offspring, as confirmed by a meta-analysis of ten studies (2992 participants, both mothers and offspring) [11]; (2) detection of enteroviruses in stools [12] and circulating antivirus neutralising antibodies [13] precedes the appearance of islet autoantibodies by several months in children at increased genetic risk for type 1 diabetes; and (3) faster progression to type 1 diabetes occurs in autoantibody-positive children with enterovirus RNA in their blood [6].

Genetic studies imply that innate responses to viruses are controlled by alleles associated with risk of type 1 diabetes. The *IFIH1* gene [14] encodes melanoma differentiation-associated protein 5 (MDA5), a helicase that recognises double-stranded RNA (dsRNA) generated during enterovirus replication, and promotes interferon, NF-κB and cytokine responses. In the presence of *IFIH1* predisposing alleles, the interferon response to viral infection of pancreatic islets is altered [15]. Interferons upregulate HLA class I molecules and this is a key feature of beta cell pathology in type 1 diabetes [16], which increases the cell's potential to present self-antigens and trigger autommunity [17]. Other genes that have an impact on viral infections, such as *PTPN2 *and *TYK2*, are linked to the genetic risk of type 1 diabetes. These genes promote endoplasmic reticulum (ER) stress, leading to impaired beta cell function and survival [18, 19]. The link between environmental factors and genetic predisposition supports the concept that genetically determined host responses to enteroviruses affect the outcome of infection and the development of type 1 diabetes. Overall, epidemiological evidence supports an association between enterovirus infection and islet autoimmunity and type 1 diabetes, whilst genetic data link disease with enteroviral infections and host responses to the virus.

## Pancreas pathology reveals low-grade, chronic enterovirus infections

Access to the pancreas at or soon after diagnosis of type 1 diabetes is rare, and so are reports that viruses can be isolated from the pancreas at this stage. In 1979, CVB4 was isolated from the pancreas of a child with recently diagnosed type 1 diabetes; the virus infected mice and caused viral protein expression in beta cells, islet inflammation, beta cell necrosis and hyperglycaemia [20]. In 2007, CVB4 infection was reported in the pancreas of three of six individuals with type 1 diabetes and, following extraction, the virus infected beta cells from pancreatic donors without diabetes [21]. However, acute, lytic, widespread viral infections have not been reported in the pancreas in type 1 diabetes (except in rare cases of the atypical fulminant type 1 diabetes [22]), regardless of whether samples were from the Exeter Archival Diabetes Biobank (EADB; autopsy pancreases) [23], the Network for Pancreatic Organ Donors with Diabetes (nPOD) [24] or the Diabetes Virus Detection Study (DiViD) [25], all of which include pancreases from individuals with newly diagnosed type 1 diabetes.

In the DiViD study, pancreas biopsies were obtained from six adults with recent-onset type 1 diabetes [26]. Using RT-PCR and sequencing, enterovirus was detected in four of these donors but in none of six non-diabetic control donors [25]. In contrast, using combined virus culture with PCR, enterovirus genome was detected in all six DiViD donors with type 1 diabetes and, when isolated, the viruses from these donors infected permissible cell cultures and were propagated between cultures [27]. The enterovirus capsid protein viral protein 1 (VP1) and marked HLA class I hyperexpression were detected in islets from all type 1 diabetes DiViD donors, compared with only *n* = 2 and *n* = 1 of the nine control donors tested, respectively [25].

Consistently across the EADB, DiViD and nPOD cohorts, VP1 is detected in a small proportion of insulin-positive, residual beta cells; within VP1^+^ cases, between 6.9% and 28.6% of residual insulin-containing islets display VP1^+^ cells and, within those, between 1.8% and 5.5% of the endocrine cells are VP1^+^ (Table 1) [21, 23–25]. This is especially true in islets with insulitis and/or hyperexpressing HLA class I molecules [16, 28] and has been found across a wide age range [23, 24]. In studies of nPOD donors, unbiased proteomic analysis has verified the presence of viral proteins, including the VP1 epitope (J. Nyalwidhe and J. Nadler, Eastern Virginia Medical School, Norfolk, VA, USA, personal communication). Studies of nPOD donors demonstrate that VP1 can be detected for about 10 years after diagnosis. Overall, the findings are consistent with the notion that low-grade, recurrent or chronic, persistent infections affect the pancreas in type 1 diabetes [29]. Conversely, the data do not support widespread acute infection and direct cytopathic effects on infected cells in type 1 diabetes.

**Table 1:** Comparison of VP1 status in three pancreas biobank cohorts (EADB, nPOD and DiViD)

Variable	EADB [23, 24]	nPOD [24]	DiViD [25]
Control/T1D donors, *n*	119/72	12/17	9/6
Age of T1D donors, mean years ± SEM	12.7 ± 1.1	25.7 ± 2.9	28.8 ± 2.1
VP1^+^ control donors, *n* (%)	12 (10)	1 (8)	2 (22)
VP1^+^ T1D donors with residual ICIs, *n* (%)	44 (61)	8 (80)^a^	6 (100)
No. of T1D islets with VP1^+^ cells/total no. of residual ICIs, (%)	77/374 (20.6)	65/227 (28.6)	42/612 (6.9)
VP1^+^ ECs in VP1^+^ T1D ICIs^b^, % ± SEM	PM: 1.76 ± 0.32 OD: 5.10 ± 0.87	OD: 5.52 ± 0.90	ND

^a^No residual insulin-containing islets in *n* = 7

^b^Source: [23]

EC, endocrine cell; ICI, insulin-containing islets; ND, no data; OD, organ donor; PM, post-mortem donor; T1D, type 1 diabetes

## Beta cells can sustain chronic enterovirus infections

Enteroviruses typically cause acute infectious diseases, dominated by poliomyelitis, childhood hand, foot and mouth disease, aseptic meningitis and acute myocarditis. However, enteroviruses can persist in certain tissues after the acute phase, as observed in the heart in myocarditis [30] and mouse pancreases [31]. Persistence is ascribed to deletion of up to 50 nucleotides from the 5′ end of the virus genome [31, 32], which reduces replication and pathogenic potential. The virus becomes non-lytic and persists for months at low levels; in beta cells, this may lead to altered gene expression, ER stress and toxicity through continued production of viral proteases that regulate host translation and transcription [33]. VP1^+^ beta cells show decreased insulin content and marked upregulation of the dsRNA sensor protein kinase R (PKR) [24]. These cells undergo translational arrest in response to PKR activation, with loss of the anti-apoptotic protein myeloid cell leukemia 1 (Mcl-1) [34]. Islet cells from individuals with newly diagnosed type 1 diabetes show increased expression of interferon response genes and the transcription factor signal transducer and activator of transcription 1 (STAT1), which is associated with hyperexpression of HLA class I molecules, demonstrated both at the protein and RNA level [16]. Once again, the impact of enteroviruses in diabetes is not simply explained by the infection per se, but rather derives from host–virus interactions, which can be chronic. Thus, it is important to consider the impact of viral infections in the context of the target tissue and host responses and the fact that available data about the role of enteroviruses in type 1 diabetes are not consistent with the conventional concept of an acute, cytopathic infection.

## The problem of linking common or slowly developing chronic infections with disease: parallels between diabetes and cancer

**Fig. 1:**
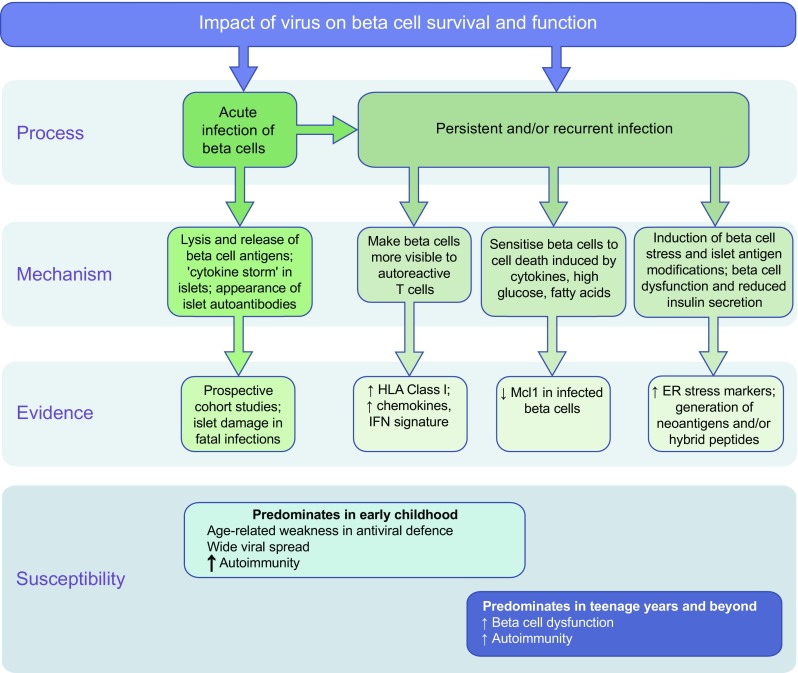
Mechanisms by which viruses may impact beta cell survival/function and contribute to type 1 diabetes pathogenesis. Evidence for each of the different proposed mechanisms is presented. Genetic susceptibility to type 1 diabetes could contribute to outcome, with specific disease-associated SNPs influencing which of the viral impact mechanisms predominate in a given individual. Furthermore, there is mounting evidence that pathways could differ depending on age at type 1 diabetes onset; although each of the pathways can occur at any age, there is evidence that autoimmunity is more pronounced in individuals with a younger age at onset, whilst beta cell dysfunction is greater in individuals who are older at onset

The difficulties associated with attempts to demonstrate an association of viruses with type 1 diabetes parallels the problems encountered when establishing causal relationships between viruses and cancer. Examples include human papillomavirus (linked with cervical or throat cancer) and hepatitis B and C viruses (linked with hepatocellular carcinoma). Proof of viral causality for these cancers was challenging because of virological and epidemiological issues, which similarly hamper the acceptance of a viral role in type 1 diabetes today. The first issue is that enterovirus infections are common in the population and have a much higher incidence than type 1 diabetes. Moreover, only certain serotypes may trigger disease [9]. Importantly, beta cells express the receptor for these serotypes in the secretory granule membrane where it may promote infection by facilitating the uptake of virus into the cell during insulin secretion [35]. Second, there is typically a long incubation period between the initial viral infection and diabetes symptoms, coupled with complex processes leading from infection to disease. Third*,* the initial infection may be asymptomatic, preventing firm establishment of the time of infection. While a high viral load is reported in virus-associated cancers, so far there has been no evidence of acute, severe enterovirus infections affecting the pancreas (or other organs) relevant to type 1 diabetes, including in studies of pancreas autopsies and biopsies from donors with new-onset diabetes. This possibility cannot be formally excluded because short-lived, acute infections may not be easily demonstrated in the pancreas for obvious reasons of timing and access; however, massive viral cytotoxicity has not been noted in beta cells. Fourth, variation of individual hosts will play a critical role in disease development; this may arise from variation in genes that modulate adaptive and innate immune responses (i.e. HLA alleles, *IFIH1*), age when infection occurs, prior immunity and the status of the immune system (Fig. 1).

Type 1 diabetes (like cancers) results from a multistage process in which viruses may influence the host immune system, stress responses and other molecular events in both target cells and cells of the immune system. The mechanisms leading to type 1 diabetes occur in the pancreas, involve pancreatic cells and cells of both the adaptive and innate immune systems, and are difficult to model experimentally. A major difference between cancer and type 1 diabetes is the association with HLA in the latter. The observation that the first appearing autoantibodies in type 1 diabetes are related to *HLA-DR4-DQ8* and to prior enterovirus infections [36] suggest that viruses may be involved in triggering beta cell autoimmunity. Thus, with some important differences as compared with cancer, the association of viral infections with type 1 diabetes needs to be evaluated in the context of a complex disease with multiple pathogenic mechanisms, some of which remain to be unveiled.

## Emerging evidence for multiple viral hits by multiple virus strains

In one DiViD type 1 diabetes donor, one enterovirus strain was detected in the pancreas, whilst a different one was found in peripheral blood mononuclear cell (PBMCs), stool and duodenum. This is consistent with a low-grade persistent infection in the pancreas (residing from the prediabetic period) plus an acute systemic infection at diagnosis (K. Dahl-Jørgensen, unpublished results). In nPOD type 1 diabetes donors, using PCR, multiple enteroviruses were detected in the pancreas of individual donors (H. Hyöty and R. Lloyd, unpublished results), suggesting that infections with more than one enterovirus can co-exist. Enterovirus infection of the pancreas may occur multiple times in an individual’s life, possibly sustained by multiple serotypes. Enterovirus infection with a given serotype induces neutralising antibodies that do not afford cross-protection against different serotypes. Even in the presence of neutralising antibodies, a second infection with the same strain may not be prevented; depending on the titres of neutralising antibodies, the second infection may lead to persistence and chronic tissue damage [37]. It is also unclear whether all or only some virus infections in the pancreas in type 1 diabetes are relevant to disease onset and its progression. Moreover, other viruses and the gut microbiome may modulate enterovirus infections (and the ensuing immune responses); it is possible that an altered or permissive microbiome or impaired intestinal barrier facilitate infection by enteroviruses [38]. All of the above suggest the hypothesis that, rather than being caused by a single, acute infection, multiple viruses (not just enteroviruses) and other environmental factors may provide multiple ‘hits’ that, over time, promote type 1 diabetes.

## How viral infections might contribute to the pathogenesis of type 1 diabetes

Viruses may trigger islet autoimmunity, potentially via molecular mimicry, inflammation, ER stress and, ultimately, host responses, such as bystander activation or suppression of T cells, which are detrimental to beta cell function and survival (Fig. 1). In terms of T cell and autoantibody responses, studies have reported cross-reactivity between islet cell autoantigens and certain viral proteins [39], yet there is no proof that cross-reactivity triggers islet autoimmunity. On the other hand, at-risk children with insulin autoantibodies have reduced ability to produce antibodies to VP1, which could facilitate enterovirus infection and persistence [7]. In addition, virus-induced inflammation and ER stress cause beta cell dysfunction and protein misfolding, which may lead to abnormal presentation of autoantigens [40–42]. Cytokine-induced ER stress enhances beta cell release of exosomes loaded with autoantigens and immuno-stimulatory chaperones, which are taken-up by antigen presenting cells (APCs) [43].

Viruses may also accelerate disease onset in genetically susceptible individuals; for example, in the Diabetes and Autoimmunity Study in the Young (DAISY), there was faster disease progression in autoantibody-positive individuals who encountered an enterovirus, as indicated by virus RNA in the blood, than those who had a negative serum enteroviral RNA test [6]. Similar observations were made in NOD mice infected with CVBs close to disease onset [44–46]. A prerequisite for faster disease onset in the NOD mouse model is the presence of a critical mass of autoreactive T cells within the islets [44, 47], which may undergo bystander activation [48].

Multiple infections may, therefore, represent iterative triggers for chronic islet autoimmunity, with diabetes symptoms becoming manifest when sufficient beta cells have been lost or have become dysfunctional [49]. In support of this, CVB4 caused beta cell dysfunction in islet grafts when human islets were transplanted into nude mice [50].

The adaptive immune responses to enteroviruses likely play a larger role in type 1 diabetes than is currently recognised. Insulitis (predominantly involving CD8^+^ T cells) and hyperexpression of HLA class I molecules in islet cells are defining features of the pancreas pathology in type 1 diabetes [16, 49] and correlate with markers of viral infection in beta cells (VP1) [16]. The immune system may launch an immune response against viral epitopes on infected beta cells, leading to beta cell destruction regardless of whether these responses are cross-reactive with autoantigens.

The relationship between beta cell destruction and antiviral immune responses is essentially unknown. A fascinating hypothesis is that viruses and infected beta cells may survive if the infected cells present viral epitopes that are cross-reactive with beta cell antigens; while cross-reactivity is traditionally considered a trigger, immune responses to cross-reactive epitopes could be regulated and, thus, not highly pathogenic, which would allow infected cells to survive and the virus to persist. So far, a small case−control study identified eight CVB3 epitopes that are presented by HLA-A*0201, but did not show an association of T cell responses with type 1 diabetes [51]. If immunisation with an enterovirus vaccine were to induce a CD8^+^ T cell response, it would be important to assess potential cross-reactivity with islet autoantigens. However, accelerated disease was not observed in NOD mice receiving a monovalent CVB vaccine [46].

Overall, the characterisation of cellular immune responses to enteroviruses in type 1 diabetes, and the relationship of such responses with autoimmune responses, remains a major area of investigation, which could yield critical information about the mechanisms by which enteroviruses contribute to pathogenesis of the disease.

## Rationale for the development of a vaccine

Vaccines would be useful tools to obtain proof of causality between type 1 diabetes and enteroviruses. Effective and safe vaccines have been developed against polioviruses and enterovirus 71, but there are no vaccines available for the enteroviruses linked to type 1 diabetes. Recent preclinical studies provided proof-of-concept that a CVB vaccine prevents viral infection and diabetes induction in mice harbouring beta cells genetically permissive to CVB infection [52]. It is critical to determine which virus serotypes are associated with type 1 diabetes in different geographical regions over various periods. Existing information is guiding the development of a polyvalent vaccine directed against CVB serotypes. Additional information is expected from on-going efforts in the Environmental Determinants of Diabetes in the Young (TEDDY) study (conducted in the USA and Europe) and the Type 1 Diabetes Prediction and Prevention (DIPP) studies (in Finland), and from the analysis of pancreas pathology.

**Fig. 2:**
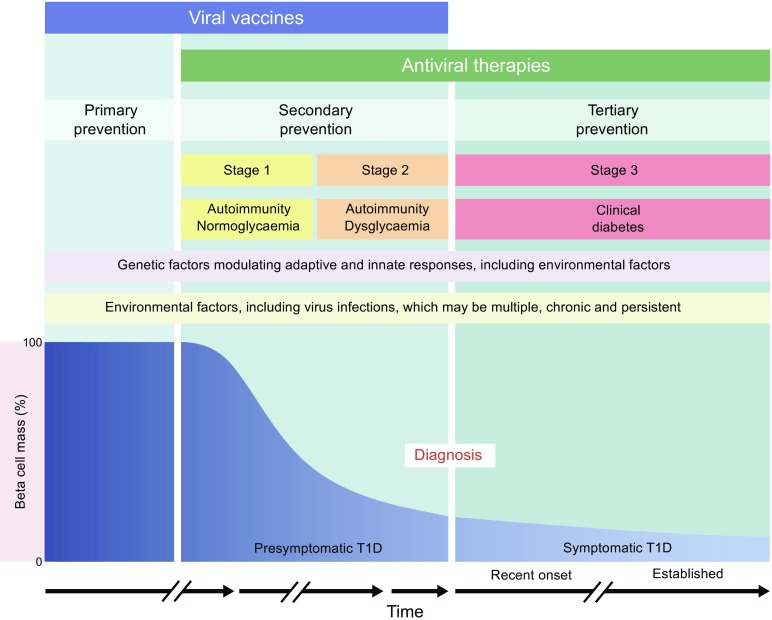
Applicability of viral vaccines and antiviral therapies to primary, secondary and tertiary prevention of type 1 diabetes, according to the disease stages described in the scientific statement of the JDRF, the Endocrine Society, and the ADA [53]. The schematic presented here is a development of the original schematic presented by Insel et al [53]. Material from this publication has been used with the permission of ADA. Copyright and all rights reserved

An enterovirus vaccine may be effective for primary and secondary prevention of type 1 diabetes, by halting the triggering of autoimmunity and blocking the onset of immune-mediated beta cell dysfunction and death that follow autoantibody conversion in presymptomatic (stage 1) type 1 diabetes [53] (Fig. 2). Mechanisms of secondary prevention with an enterovirus vaccine could include: (1) prevention of consecutive islet infections; (2) prevention of a systemic infection that either reactivates immune memory resulting in an islet-targeted immune response or increases the circulating cytokines and inflammation that generate a relapse or augmentation of islet autoimmunity; and/or (3) limitation of an intestinal enterovirus infection, which might otherwise accelerate the onset of symptomatic disease by altering intestinal permeability. A vaccine against enteroviruses does not directly address the potential contribution of other viruses to type 1 diabetes onset and progression, yet it is a pragmatic choice based on the more extensive association of enteroviruses with islet autoimmunity and type 1 diabetes.

A potential alternative to vaccination, testable in secondary and tertiary (new-onset; Fig. 2) prevention trials, is treatment with antiviral drugs to eliminate an established chronic infection of the pancreas. One such clinical trial (Diabetes Virus Detection and Intervention Trial [DiViDInt], launched in 2018, will enrol individuals with newly diagnosed type 1 diabetes to a randomised, placebo-controlled double-blinded trial to test a combination of two antiviral drugs (pleconaril and ribavirin). Antivirals were effective in vitro in pancreatic cell lines with persistent enterovirus infections [54]. We have recently confirmed this using a wider panel of antiviral drugs (H. Hyöty et al, unpublished results). Antivirals are effective in other chronic infections, such as hepatitis B and C [55, 56]. The endpoint of this study will be preservation of beta cell function; viral markers also may be explored in stools, blood and saliva [57] but it is presently not known that these would reflect changes in pancreatic infections. Based on the evidence discussed so far, both antivirals and vaccination strategies could play a key role in understanding the role of enteroviruses in type 1 diabetes and could impact on disease risk and clinical course.

## Cost-effectiveness of viral vaccines in type 1 diabetes

The commercial viability of a viral vaccine for therapy of type 1 diabetes needs to be formally evaluated based on its efficacy in target populations and the overall risk and lifetime cost of the disease. In addition to the rising disease incidence, the cost of treatment for stage 3, symptomatic clinical disease (e.g. insulin, pumps, glucose monitors) is increasing. A vaccine could reduce other enterovirus comorbidities (e.g. aseptic meningitis, myocarditis or hand, foot and mouth disease), most of which are more prevalent than type 1 diabetes. Persistent enterovirus infection and antiviral tissue responses also been detected in thyroid tissue from individuals with have newly diagnosed Graves’ disease [58], Hashimoto’s thyroiditis [59], and in those with pericarditis and myocarditis [30]. Thus, it may be possible to include other health outcomes in vaccination protocols, thereby increasing cost-effectiveness. Moreover, individuals with type 1 diabetes may suffer from comorbid coeliac disease and other autoimmune diseases [60, 61], which could conceivably be alleviated by enterovirus vaccination. Although initial enteroviral vaccine trials will probably target infants with high-to-moderate risk of type 1 diabetes based on HLA genotype, the most cost-effective approach would be universal infant immunisation with the double benefit of avoiding the cost of neonatal HLA genotyping while preventing enterovirus diseases in a greater number of children.

## Conclusions

In recent years, a combination of epidemiological, histological and animal studies has strengthened the evidence for a role for enteroviruses in type 1 diabetes. Because of the limitations of epidemiology studies, the long incubation period between the onset of islet autoimmunity and symptomatic type 1 diabetes, and the complexity of a potential role of both acute and chronic enterovirus infection in the disease pathogenesis, definitive proof of a causal role for enteroviruses in type 1 diabetes requires further investigation. Specifically, it should be demonstrated that prevention of enteroviral infections prevents type 1 diabetes. An enteroviral vaccine, and/or antivirals, can help to fulfil this goal. The recent announcement that a CVB vaccine will be developed for type 1 diabetes therapy substantiates this claim [62]. If this vaccine can prevent type 1 diabetes development, even in a proportion of cases, this would dramatically impact the health and economic burden of the disease. Efficacy may be tested initially in shorter trials using surrogate endpoints, such as progression to stage 1 disease, or progression from stage 1 to 2 or stage 2 to 3. The cost-effectiveness and early prognostic biomarkers for type 1 diabetes remain understudied and will play a critical role in determining the utility of viral vaccines. Only a rigorous evaluation of the outcomes of vaccination and antiviral therapies can provide the ultimate proof that viruses are causally associated with type 1 diabetes and could help address the mechanisms by which viruses play a role in development of the disease.

## Abbreviations

CVB: Coxsackievirus B
DiViD: Diabetes Virus Detection Study
dsRNA: Double-stranded RNA
EADB: Exeter Archival Diabetes Biobank
ER: Endoplasmic reticulum
nPOD: Network for Pancreatic Organ Donors with Diabetes
PKR: Protein kinase R
VP1: Viral protein 1
